# Complete Genome Sequence of the Encephalomyelitic *Burkholderia pseudomallei* Strain MSHR305

**DOI:** 10.1128/genomeA.00656-13

**Published:** 2013-08-22

**Authors:** Joshua K. Stone, Shannon L. Johnson, David C. Bruce, J. Chris Detter, Mark Mayo, Bart J. Currie, H. Carl Gelhaus, Paul Keim, Apichai Tuanyok

**Affiliations:** Department of Biological Sciences, Northern Arizona University, Flagstaff, Arizona, USAa; Los Alamos National Laboratory, Los Alamos, New Mexico, USAb; Menzies School of Health Research, Charles Darwin University, Darwin, Northern Territory, Australiac; Battelle Memorial Institute, Columbus, Ohio, USAd

## Abstract

We describe the complete genome sequence of *Burkholderia pseudomallei* MSHR305, a clinical isolate taken from a fatal encephalomyelitis case, a rare form of melioidosis. This sequence will be used for comparisons to identify the genes that are involved in neurological cases.

## GENOME ANNOUNCEMENT

*Burkholderia pseudomallei* is a Gram-negative saprophytic bacterium endemic to southeast Asia and northern Australia (1). It is the etiological agent of melioidosis, a disease that can present in numerous forms, from cutaneous to pneumonia to septicemia (2). Neurological cases occur rarely, in only 3 to 4% of patients, and in murine models, inhalation led to direct brain infection, presumably through the nasal mucosa via the olfactory nerve (2–4). *B. pseudomallei* strain MSHR305 was isolated in 1994 during an autopsy of a fatal melioidosis encephalomyelitis case at the Royal Darwin Hospital, Northern Territory, Australia, and was previously sequenced to 36 contigs (5).

DNA sequencing was performed by the Los Alamos National Laboratory (LANL) Genome Science Group using Illumina and PacBio technologies (6, 7). Short-insert paired-end Illumina libraries yielded 27,714,112 reads totaling 27.7 Mbp, and long-insert libraries generated 920,819 paired reads totaling 159 Mbp. The PacBio library generated 260,870 subreads for a total of 570 Mbp. The Illumina short-insert and long-insert paired data were assembled together using Newbler version 2.6. The Newbler consensus sequences were computationally shredded into 2-kbp overlapping short reads (shreds). The Illumina data were also assembled with Velvet version 1.2.08 (8), and the consensus sequence was shredded into 1.5-kbp overlapping shards. The draft data from all platforms were assembled together with AllPaths, version 39750, and the consensus sequence was shredded into 10-kbp overlapping shreds. We integrated consensus shreds from Newbler, Velvet, and AllPaths along with a subset of read pairs from the Illumina long-insert library using parallel Phrap version SPS-4.24 (High Performance Software, LLC). Gap closure was accomplished using PacBio consensus sequences.

The final assembly consisted of two chromosomes of lengths 4,054,138 and 3,373,904 bp, with average coverages of 287× and 79.2× for the Illumina and PacBio reads, respectively, and 6,209 total predicted genes. A comparison to the prototypic strain *B. pseudomallei* K96243 and to *B. pseudomallei* MSHR668, an isolate from a nonfatal encephalomyelitis case (5), revealed the presence of a hemolysin and activator protein on chromosome 1 in MSHR305 and MSHR668 that is not present in K96243. However, BLAST searches reveal that these proteins are in most *B. pseudomallei* strains. With the results of Owen et al. (4), it appears that most strains have the capacity to cause neurological melioidosis if the bacteria are inhaled and travel directly to the brain. We note that MSHR305 is one of the six priority *B. pseudomallei* strains that were selected as challenge materials for medical countermeasure (MCM) efficacy testing by the Biomedical Advanced Research and Development Authority (BARDA) (9).

### Nucleotide sequence accession numbers. 

This whole-genome sequencing project has been deposited at GenBank under the accession no. CP006469 and CP006470. The version described in this paper is the first version.
